# Artificial intelligence promotes the diagnosis and screening of diabetic retinopathy

**DOI:** 10.3389/fendo.2022.946915

**Published:** 2022-09-29

**Authors:** Xuan Huang, Hui Wang, Chongyang She, Jing Feng, Xuhui Liu, Xiaofeng Hu, Li Chen, Yong Tao

**Affiliations:** ^1^ Department of Ophthalmology, Beijing Chaoyang Hospital, Capital Medical University, Beijing, China; ^2^ Medical Research Center, Beijing Chaoyang Hospital, Capital Medical University, Beijing, China

**Keywords:** diabetic retinopathy, artificial intelligence, classification, segmentation, diagnosis, screening, prediction

## Abstract

Deep learning evolves into a new form of machine learning technology that is classified under artificial intelligence (AI), which has substantial potential for large-scale healthcare screening and may allow the determination of the most appropriate specific treatment for individual patients. Recent developments in diagnostic technologies facilitated studies on retinal conditions and ocular disease in metabolism and endocrinology. Globally, diabetic retinopathy (DR) is regarded as a major cause of vision loss. Deep learning systems are effective and accurate in the detection of DR from digital fundus photographs or optical coherence tomography. Thus, using AI techniques, systems with high accuracy and efficiency can be developed for diagnosing and screening DR at an early stage and without the resources that are only accessible in special clinics. Deep learning enables early diagnosis with high specificity and sensitivity, which makes decisions based on minimally handcrafted features paving the way for personalized DR progression real-time monitoring and in-time ophthalmic or endocrine therapies. This review will discuss cutting-edge AI algorithms, the automated detecting systems of DR stage grading and feature segmentation, the prediction of DR outcomes and therapeutics, and the ophthalmic indications of other systemic diseases revealed by AI.

## Introduction

Diabetic retinopathy (DR), an eye disease that is associated with severe visual impairment, is the leading cause of blindness in diabetics (1). DR occurrence is attributed to chronic high blood glucose levels that lead to retinal capillary damage hindering light perception and signal transmission. DR, whose incidence is high in the working-age population, prevails all over the world and is estimated to reach 191 million cases by 2030 (2). Despite the progression of DR leading to blindness, the detection of DR in early stages is challenging due to its imperceptible visual signs. Hereby, regular screening and early diagnosis can reduce the visual loss risk by 57.0% as well as treatment costs (2). The screening tests for DR (usually retinal photography) are safe, simple, acceptable, and benefit-validated by many longitudinal studies (3, 4). Efficacious treatments, such as the intravitreal injections of antivascular endothelial growth factor (VEGF) agents and laser therapies for severe DR, are available for patients identified through early detection. However, many countries lack the resources for nationwide screening. Therefore, a simple and scalable community screening solution is needed with insufficiently trained ophthalmology. As an important public healthcare problem, DR meets all the criteria for screening that has long been recommended by many international societies (5).

The new technology based on artificial intelligence (AI), which permits to implement large-scale detection and personalized predictive models, is shifting screening strategies and enhancing the cost-effectiveness of screening (6). Advances in computer-aided diagnostic (CAD) techniques in modern ophthalmology are efficient in saving time and human resources as well as costs for routine DR screening and are associated with low diagnostic errors (7). CAD has also been shown to effectively handle the rising number of referable DR patients and diagnosis of DR at an early stage with few sight- threatening effects (8). Variations in these techniques are based on different non-invasive imaging systems, including ultrawide-field fundus (UWF), optical coherence tomography (OCT), OCT angiography (OCTA), standard 45° fundus photography, and even the camera equipped in smartphones applied to in-time DR screening (9). Machine learning (ML)–based algorithms, especially deep learning (DL), are not only efficient for the detection, localization, and quantification of pathological features mimicking the path of the human brain for target recognition in DR but could also diagnose or classify DR stages from patterns recognized independently, by unsupervised convolutional neural networks (CNNs) (10). Although AI-based retinal analysis methods widely differ in their applicability, reliability, and interpretability in different diseases and datasets, recently, fully automated AI-based systems have been further developed and initially approved for DR screening (11, 12).

In this review, we will analyze the applications of ML/DL comprehensively for the screening and diagnostic grading of DR and the mechanistic features of DR progression revealed by AI, as well as the AI guidance of the prognosis and therapy systems with an automated identification of disease activity, recurrence, and therapeutic effect evaluation. In addition, the use of retinal examination to establish the risks for other diseases will be commented, thus expanding the role in the diagnosis and screening of DR.

## Development of artificial intelligence algorithm

**Figure 1:**
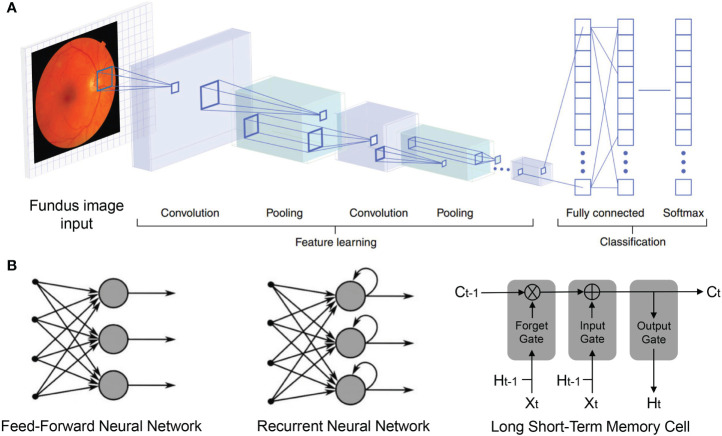
Deep neural network structures. **(A)** Convolutional neural network (CNN) imaging flow: Fundus images are input and sequentially transformed by convolution, pooling, and fully connected layers, into flattened vectors. Output vector (Softmax layer) elements denote the probabilities for disease presence. In training, lower layers (left) learn features to influence the high-level representations (right), by which internal network layer parameters are iteratively adjusted to enhance accuracy. **(B)** General architectures of deep learning models in mainstream.

With increased computational power and the availability of new datasets, DL has experienced a dramatic resurgence recently as a subfield of machine learning. The diagnosis and therapeutics of DR have benefited greatly from DL owing to the volume of big data and the increasing application of ophthalmic devices as well as digital record systems. Scaling to large datasets, DL models show successive improvements with more data, which enable them to outperform many classical ML approaches. The majority of available models are trained by supervised learning, whereby datasets have data points (e.g., fundus lesion) as input and the matched data labels (e.g., mild or severe) as output. Commonly first, the algorithm uses the large data amounts to learn the natural features of statistics in images, like curves, straight lines, and colorations among others. Additionally, in the second step, in order to differentiate among diagnostic cases, the higher-level layers of algorithms are involved to be retrained. Moreover, the identification of the specific image parts that correspond to specific diagnostic objects is based on target detecting and segmentation algorithms. The image data are taken as input by CNN approaches that iteratively warp the pixels *via* multiple convolutional and non-linear operations until the original raw data matrix is transmuted into a probability distribution over potential image classes (13) (**Figure 1**). Aside from the CNN as a feed-forward neural network designed to process data with network structures, the recurrent neural network (RNN) is a specialized neural network for processing sequential data, such as time series. In addition, the long short-term memory (LSTM) algorithm is a variant of RNN that aims to prevent the vanishing gradient problem in RNN by using a memory gating mechanism (**Figure 1**). They all have contributed to the application of AI across the human lifespan (14).

Image-level diagnostics could employ CNN-based approaches, including Inception V3, Xception, InceptionResNet V2, ResNeXt101, and NASNetLarge, applying transfer learning from ImageNet. We have proposed multiple algorithms for DR detection and grading based on deep ensemble learning and attention mechanisms to integrate the classic algorithms (15). Relative to the traditional single network model detection algorithm, the area under the receiver operating characteristic curve (AUC), accuracy, and recall of the suggested approach are respectively improved to 95%, 92%, and 92%, proving the optimization and adaptability of fusion algorithms for fundus photographs. For OCT images, we have also applied a fusion network algorithm to the retinal lesion classification of choroidal neovascularization (CNV), diabetic macular edema (DME), drusen, and normal groups. The result showed that the developed fusion algorithm can significantly improve the performance of classifiers compared to traditional algorithms while providing a powerful tool and theoretical support to assist with the diagnosis of retinal OCT images (16).

For a long time, two types of learning tasks have been used to construct AI models: supervised learning with training from known patterns and labeled input data commonly referred to as ground truths and unsupervised learning from unknown patterns without labeled input data. Unsupervised learning has limited power up to date, while an automatic supervised solution is challenging due to the requirement of a large amount of training data and the laborious annotations for medical images. Therefore, novel self-supervised learning frameworks for retinal disease diagnosis are presented nowadays (17–20). This so-called unsupervised visual representation learning derives labels from a co-occurring input to relate information, thus reducing the annotation efforts by learning the visual features from the unlabeled images. For instance, Xiaomeng Li et al. developed a self-supervised method surpassing the supervised baseline for the classification of pathologic myopia (PM) and age-associated macular degeneration (AMD) (21).

Another solution to the training need for large, criterion standard–annotated retinal datasets is few-shot deep learning. This algorithm aims to learn from a relatively low number of training data, which is beneficial for clinical situations involving rare retinal diseases or when addressing potential bias resulting from data that may not adequately represent certain groups for training. Tae Keun Yoo et al. demonstrated that few-shot learning using a generative adversarial network (GAN) could improve the applicability of DL in the OCT diagnosis of rare retinal diseases (22). The potential benefits of using low-shot methods for AI retinal diagnostics due to a limited number of annotated training retinal images have been confirmed as feasible (23).

## Automated detection and classification of diabetic retinopathy

New ML and DL approaches are viable for automated DR diagnosis. Various performance metrics, including accuracy, specificity, AUC, sensitivity or recall, precision, F1 score, and Kappa score, have been used for the evaluation of grading, namely, classification performance. The International Council of Ophthalmology classification for DR considers five retinopathy stages (none, non-proliferative: mild, moderate, severe, and proliferative), while DME is classified as no DME, non-center involving DME, and center-involving DME (6). These clinical standards are widely used in the practice of AI-based implementation.

In 2016, Gulshan et al. (24) established a potent DL algorithm for DR assessment. Approximately 0.13 million images from two public databases (EyePAC-1 and Messidor-2) were used to train their model. As a result, 0.97–0.99 AUC values were acquired from tests using two distinct datasets to detect referable (moderate or worse) DR achieving physician-level accuracy. To facilitate translation and develop DL for clinical applications using multiform retinal images of inhomogeneous qualities from different camera types in the representative screening populations sensitive to DR, Ting D.S.W. et al. (25) built a diagnostic model for DR based on larger datasets that consist of approximately 0.5 million images from a multiethnic community. In the primary validation dataset (14, 880 patients; 71, 896 images), the automated model had high sensitivity (90.5, 100, 96.4, 93.2%) and specificity (91.6, 91.1, 87.2, 88.7%) for distinguishing vision-threatening DR from referable DR and for the identification of related eye diseases, such as referable glaucoma and referable AMD. Based on a solid evaluation of retinal images by trained professional graders, the performance of this model was comparable to that of the current first class. Using various reference standards to assess DR by professional graders, retinal specialists, and optometrists, there was fair consistency in the 10 external validation datasets of multiethnicities and diverse settings.

Similarly, Abramoff et al. (26) developed an automated system for DR detection with a sensitivity of 96.8% and specificity of 59.4% using the CNN algorithm on a publicly available dataset (Messidor-2), considered as pathbreaking work in this field. Subsequently, studies have further assessed the suitability of the DL technology for DR detection (27) and grading (28, 29). Gargeya and Leng (27) reported optimal DL diagnostic performance in the detection of DR achieving a 0.97 AUC with a specificity and sensitivity of 98% and 94%, respectively, based on two publicly available databases (E-Ophtha and Messidor-2). Likewise, Philip et al. (30) documented a sensitivity and specificity of 86.2% and 76.8%, respectively, for predicting disease versus no disease on their own dataset of 14, 406 images of DR screening. High-quality datasets with precise DR grading are essential to developing a DL system for automated detection and classification (31), so we present an overview of the available and high-level datasets that are public open access (OA) or access upon request (AUR) (**Table 1**).

**Table 1:** Datasets for diabetic retinopathy (DR) detection, segmentation, and grading.

Dataset	No. of images	No. of subjects		Device used	Access		Country	Year	Type	Remarks
**DRIVE (** 32 **)**	40	400		Canon CR5 non-mydriatic 3CCD camera with a 45° FOV	OA		Netherlands	2004	CFP	Retinal vessel segmentation and ophthalmic diseases
**DIARETDB0 (** 33 **)**	130	NA		50° FOV DFC	OA		Finland	2006	CFP	DR detection and grading
**DIARETDB1 (** 34 **)**	89	NA		50° FOV DFC	OA		Finland	2007	CFP	DR detection and grading
**HEI-MED (** 35 **)**	169	910		Visucam PRO fundus camera (Zeiss, Germany)	OA		USA	2010	CFP	DR detection and grading
**DRiDB (** 36 **)**	50	NA		Zeiss Visucam 200 DFC at a 45° FOV	OA		Croatia	2013	CFP	DR grading
**E-Ophtha (** 37 **)**	463	NA		NA	OA		France	2013	CFP	Lesion detection
**DRIMDB (** 38 **)**	216	NA		CF-60UVi fundus camera (Canon)	OA		Turkey	2014	CFP	DR detection and grading
**MESSIDOR 1 (** 39 **)**	1,200	NA		Topcon TRC NW6 non-mydriatic at a 45° FOV	OA		France	2014	CFP	DR and DME grading
**Srinivasan (** 40 **)**	3,231	45	SD-OCT (Heidelberg Engineering, Germany)			OA	USA	2014	OCT	DR detection and grading, DME, and AMD
**EyePACS (** 41 **)**	88,702	NA	Centervue DRS (Italy), Optovue iCam (USA), Canon CR1/DGi/CR2 and Topcon NW			OA	USA	2015	CFP	DR grading
**JICHI DR (** 42 **)**	9,939	2,740	AFC-230 fundus camera (Nidek)			OA	Japan	2017	CFP	DR grading
**Rotterdam Ophthalmic Data Repository DR (** 43 **)**	1,120	70	TRC-NW65 non-mydriatic DFC (Topcon)			OA	Netherlands	2017	CFP	DR detection
**IDRID (** 44 **)**	516	NA	NA			OA	India	2018	CFP	DR grading and lesion
**OCTID (** 45 **)**	500	NA	Cirrus HD-OCT machine (Carl Zeiss Meditec)			OA	Multiethnic	2018	OCT	DR, AMD, and hypertension
**APTOS (** 46 **)**	5,590	NA	DFC			OA	India	2019	CFP	DR grading
**OCTAGON (** 47 **)**	213	213	DRI OCT Triton (Topcon)			AUR	Spain	2019	OCTA	DR detection
**ODIR-2019 (** 48 **)**	8,000	5,000	DFC (Canon, ZEISS, Kowa)			OA	China	2019	CFP	DR, AMD, glaucoma, and hypertension
**OIA-DDR (** 49 **)**	13,673	9,598	NA			OA	China	2019	CFP	DR grading and lesion segmentation
**FGADR (** 50 **)**	2,842	NA	NA			OA	UAE	2021	CFP	DR and DME grading
**MESSIDOR 2 (** 26 **)**	1,748	874	Topcon TRC NW6 non-mydriatic at a 45° FOV			AUR	France	Update	CFP	DR and DME grading

DFC, digital fundus camera; OA, open access; FOV, field of view; DR, diabetic retinopathy; DME, diabetic macular edema; AMD, age-related macular degeneration; AUR, access upon request; CFP, color fundus photography; OCT, optical coherence tomography; OCTA, OCT angiography; NA, not available.

Conventional fundus photography used by most studies takes the images of the macula area and optic nerve with a field of view (FOV) between 20∘ and 50∘. Although conventional FOV covers the vital region of interest for DR diagnosis and detection, there is still a large portion of uncaptured retinal surface that also matters. Takahashi et al. (42) used four-field non-mydriatic 45° fundus images to integrate into a wide retinal area for DR stage grading through a DL algorithm. With regard to DR grading, the four-field fundus photography exhibited a better performance compared to single-field conventional fundus photography. Nevertheless, four-field fundus photography in practice is laborious and time consuming, which limits its feasibility. Due to retinal imaging technology advances, a new fundus photography expanding to 200° of retinal surface images in a single shot called UWF has made a figure (51), thus providing the peripheral and posterior pole retinal region. Actually, UWF retinal images, such as UWF fluorescein angiography, have been widely used in DR diagnosis as well as treatment, attaining peripheral ischemic and neovascularization areas (52). For instance, detecting proliferative DR (PDR) *via* the DL algorithm using UWF fundus photography was proven effective by Nagasawa et al. (53).

With its unprecedented resolution, OCT permits the non-invasive visualization of the fine retinal structures. As one of the most dominant *in vivo* diagnostic tools in modern ophthalmology, spectral domain (SD)–OCT is the gold standard approach for the diagnostic imaging of macular diseases, including DME and CNV. A model based on the CNN using OCT images was shown to be able to effectively distinguish cases with advanced AMD or DME that need timely treatment, from less severe cases (54). By contrast, AI performed as well as six specialists who made ground-truth referrals using the same scans. Similarly, Chan et al. (55) integrated several DL architectures for the automatic classification of normal and DME through OCT images from a screening program in Singapore called SIDRP, thereby yielding an accuracy of 93.75%. Based on SIDRP, one of the largest DR datasets around the world, Alsaih et al. (56) compared the kinds of mainstream ML and figured out the best one to detect DME with a sensitivity and specificity of 87.5% and 87.5%, respectively. Additionally, Gerendas et al. (57) found the potential of ML/DL in the prognosis of DME patients’ best corrected visual acuity (BCVA) by OCT.

## Mechanistic interpretation of diabetic retinopathy features by artificial intelligence

AI application performing at an expert level without information on how the AI system make its decision cannot be considered as sufficient to apply. The ML/DL uses multiple representation levels to assess every retinal image operating as a black-box model without showing the actual DR lesions (e.g., microaneurysms and retinal hemorrhages). Such black-box issues may affect their clinical use negatively. Therefore, the mechanistic interpretation of DR features by AI is necessary and helpful for both clinical application and etiology research in depth. These features can likely be the contour or shape of the optic disc and tortuosity or caliber of the retinal vessels, which indicate the mechanism of certain disease progression.

**Figure 2:**
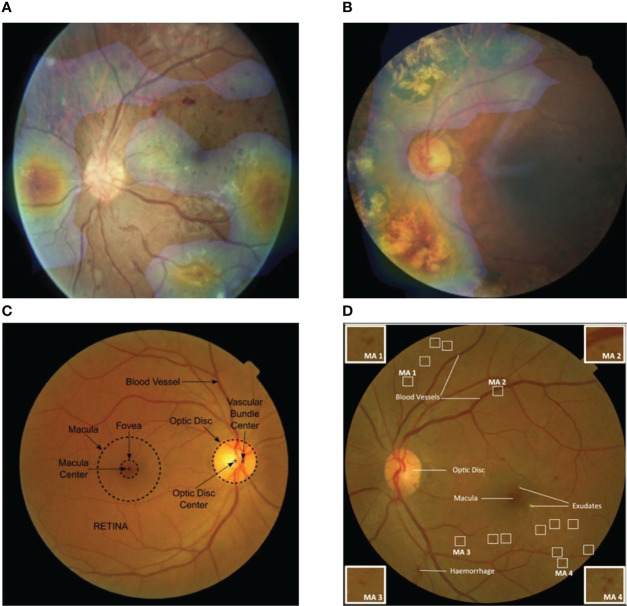
Visualization features generated automatically from color fundus photography. **(A)** Fundus heat map overlaid on a fundus image, pathologic regions of interest are in temporal and nasal quadrants as shown. **(B)** Pathologic findings are distributed in lower and upper-left quadrants as highlighted. **(C)** General anatomic landmarks for orientation in retina are labeled automatically. **(D)** Relevant pathologic structures: hemorrhage, exudates, and microaneurysms are shown. Image patches at four corners display the representative features of microaneurysm changes detected by artificial intelligence (AI) [Adapted from Ursula Schmidt-Erfurth et al. (10)].

For models based on digital fundus photography, we reviewed the general segmentation approaches of the DR lesions such as microaneurysm, hard exudate, intraretinal hemorrhage, vitreous hemorrhage, preretinal hemorrhage, neovascularization, cotton wool spots, intraretinal microvascular abnormalities, and venous beads. At the end of the network, a convolutional visualization layer was used to illustrate the prognostic regions of the fundus for DR diagnosis (27) (**Figures 2A, B**). The retinal landmarks for this mechanistic interpretation are large retinal vessels, optic discs, and sometimes foveal location as they can be found equally in each fundus image (58, 59) (**Figures 2C, D**). Ali Shah et al. (60) could detect microaneurysms in color, with Hessian and curvelet-based feature extraction achieving a sensitivity of 48.2%. Huang et al. (61) used the extreme learning machine (ELM) to localize neovascularization. They made use of standard deviation, differential invariant, Gabor, and anisotropic filters with a final classifier using ELM. Together with the support vector machine (SVM), this network performed well and resulted in lower computational time applicable to run on a personal computer (PC) or smartphone. For segmentation tasks, the preprocessing step conformed to a central rule that had direct effects on outputs. Variations in preprocessing techniques were dependent on the dataset quality and lesion type. Orlando et al. (62) combined the deep CNN (DCNN) with the manually designed features by means of illumination correction, color equalization, and Contrast Limited Adaptive Histogram Equalization (CLAHE) contrast enhancement. These high-dimensional feature vectors were fed into a random forest classifier for DR lesion detection, thus achieving an AUC score of 0.93, which was equivalent to other DCNN models (63, 64). The DCNN applied to fundus images could clearly show the lesions on retinal surfaces. For instance, Lam et al. (65) have implemented a state-of-the-art DCNN to identify DR lesions in image patches *via* VGG16, AlexNet, GoogleNet, ResNet, and Inception V3. They achieved 98.0% accuracy based on 243 fundus images from EyePACS. Wang et al. (66) have also used Inception V3 as a feature map and FCN-32s as the segmentation part. As a result, they found the sensitivity values of 60.7%, 49.5%, 28.3%, 36.3%, 57.3%, 8.7%, 79.8%, and 16.4% over preretinal hemorrhage, exudate, vitreous hemorrhage, neovascularization, cotton wool spots, fibrous proliferation, intraretinal hemorrhage, and microaneurysm, respectively. Similarly, Quellec et al. (63) focused on four lesions, i.e., cotton wool spots, exudate, hemorrhage, and microaneurysm using a predefined DCNN and reported the values of 62.4%, 52.2%, 44.9%, and 31.6% over cotton wool spots, exudate, hemorrhage, and microaneurysm for sensitivity, respectively. In comparison, this model showed slightly better performance for cotton wool spots and microaneurysm than that of Wang et al (66), while Wang et al.’s performed better in hemorrhage detection.

**Figure 3:**
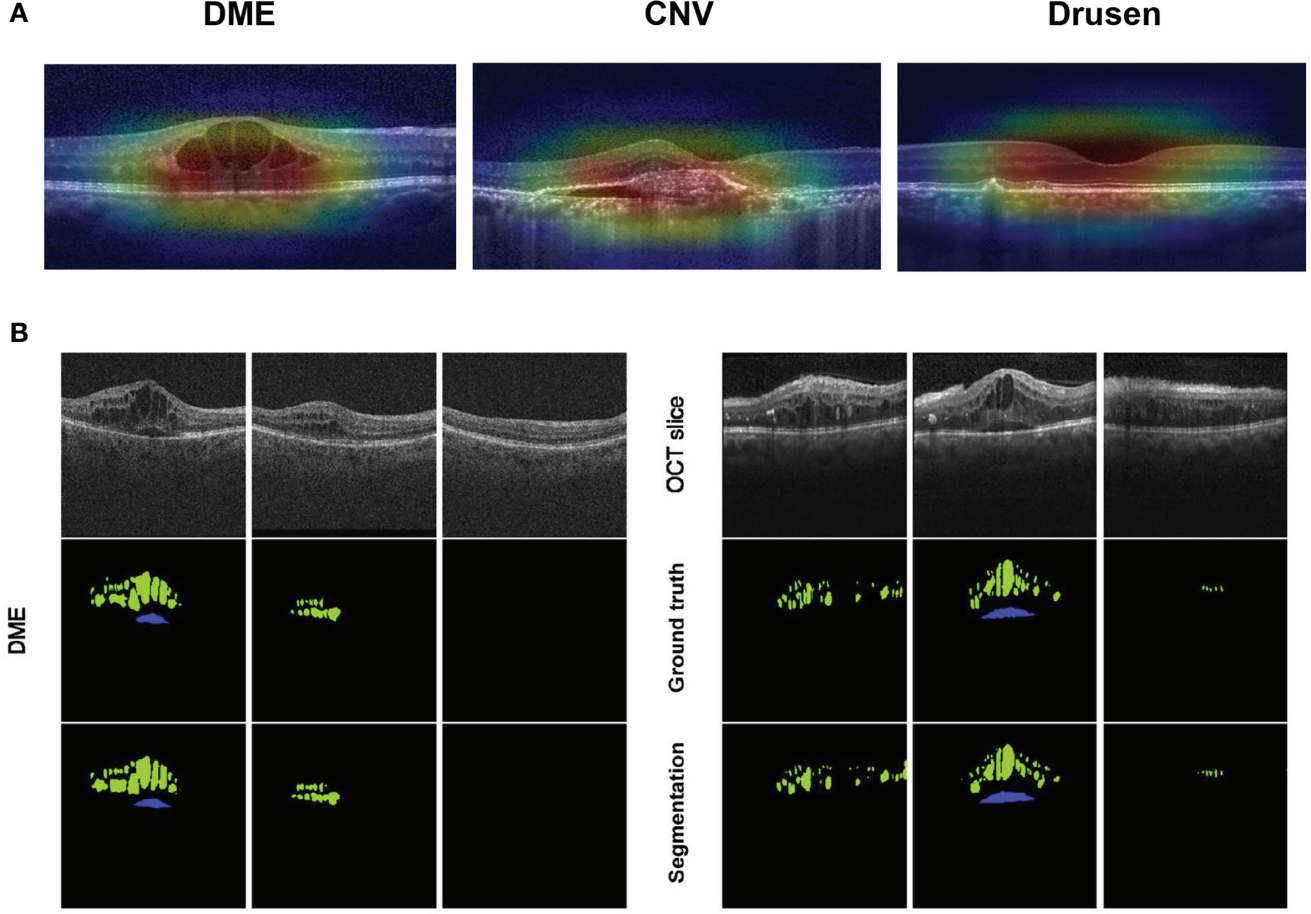
Visualization features generated automatically from optical coherence tomography (OCT). **(A)** Feature areas of pathology in diabetic macular edema (DME), choroidal neovascularization, and drusen are highlighted, superimposed on the input image to show the areas that the AI model considered as vital in a diagnosis. **(B)** Segmentation findings of DME on OCT scans acquired with Cirrus (left) or Spectralis (right) devices: the upper row shows OCT raw slices; the middle row shows manual labels by certified graders considered as ground truth; the lower row shows automated results segmented by AI. (IRF, intraretinal cystoid fluid in green; SRF, subretinal fluid in blue) [Adapted from Schlegl et al., 2018 (68)].

For OCT-based models, fovea detection is of great significance as it can also be used for orientation, especially in DME (67). Kermany et al. (54) performed an occlusion test to determine areas in the OCT image that contribute most to the decision of the neural network for DME and AMD (**Figure 3**). In OCT, the detection of intra- and subretinal macular fluid (IRF and SRF) is most applicable for exudative diseases, including DME (68) (**Figure 3**). IRF or SRF may be essential for the determination of disease activities from OCT scans and initial diagnosis of whether there is disease activity as binary. Combining fundus photographs and OCT images, Holmberg et al. (69) suggested a retinal layer extraction pipeline to assess retinal thickness, using segmentation algorithm Unet for OCT and self-supervised ResNet50 for fundus. Based on OCTA images, Yukun Guo et al. (70) used DCNNs to segment avascular zones and achieved 87.0% accuracy for mild-to-moderate DR and 76.0% accuracy for severe DR. Furthermore, Hecht et al. (71) developed ML algorithms to build a predictive classifier to diagnose DME and diabetic cystoid macular edema using SD-OCT. In order to confirm a diabetic etiology, ME pattern, hard exudates, subretinal fluid, hyperreflective foci, and cyst location within retinal layers are differentiated by the developed algorithm, resulting in a specificity of 95%, sensitivity of 96%, and AUC of 0.937. Thus, a clinical decision flowchart for uncertainty cases may support intravitreal injections rather than topical treatment.

These approaches greatly facilitate the clinical understanding of DR. In classification, real-word trust in the performance of AI and identification of probable model biases are established when physicians know which discriminative features informed decision-making. In prediction, establishing the role of individual predictive factors in AI will elucidate it further on the underlying pathophysiology. Owing to the mechanistic interpretation of DR features by AI or telemedical CAD, the black box between imaging and diagnosis becomes more and more transparent, revealing the etiological mechanism of DR. Visual explanation techniques such as Grad-CAM and Integrated Gradients are applied to generate saliency maps in order to enhance the interpretability of the algorithms. Using different methods for feature weight calculation, they generate color heat maps that highlight regions that play a critical role in the classification judgment. By visualizing the saliency maps, the contribution of different regions in the image to the final prediction could help us understand the underlying associations between ocular features and DR indicating the mechanism of disease. For DR grading, a novel multiresolution network was developed to focus on the lesion regions, providing a lesion activation map with lesion consistency as an additional evidence for clinical diagnosis (72).

## Prognosis and therapeutics guided by artificial intelligence

With respect to pattern recognition in prior data, AI methods can be used to predict the future like an experienced physician. Therefore, AI has the ability to improve the quality of care for DR patients by informing optimal therapies and to reduce healthcare costs by managing treatment prognosis. Major prediction objectives include not only functional outcomes posttherapy but also the future natural course of DR progression. Of note, AI produces and acquires knowledge that can be reproduced and accessed from data more effectively than the majority of experienced experts. Thus, clinicians are empowered to access and use prior experience from hundreds of thousands of previous cases by the ML/DL model to inform optimal treatment.

Although intravitreal anti-VEGF therapy has generated good results for the last decades and is deemed as one of the most promising medical interventions in retina, it is sometimes costly with the ever-growing number of implementations and faces challenges in the therapeutic schedule without a clear indication of benefit limiting its clinical adoption. Prahs et al. (73) trained a DCNN for the prediction of anti-VEGF indications based on central retinal OCT scans without human interventions, thus offering the clinician support in the decision-making of whether anti-VEGF is necessary or not. In the situation of DME, the implementation of anti-VEGF therapy is recommended to treat only if the intraocular fluid remains stable (74). Anti-VEGF agents can also be used to treat pigment epithelial detachment (PED) if the volume of fluid shows active growth (75, 76). Moreover, investigators propose various roles for different fluid types. For instance, IRF is a retreatment indication, while up to a definite cutoff (200 μm in height at the foveal center), but as to SRF, it may not be so (77). For these models, microstructural alterations in retina are necessary to be analyzed using efficient AI processing beyond manpower. The pathological quantification of images may also be crucial for prognoses as various biomarkers exhibit tight correlations with visual acuity and vision outcomes (78).

Reasonable retreatment intervals are important to manage healthcare effectively. As for intravitreal therapy, there is a need to achieve complete disease control while, as much as possible, avoiding the potential anti-VEGF therapy-associated morbidity including endophthalmitis (79). AI approaches will make a difference in predictive models to inform efficient treatment resolving the dilemma. Theoretically, images and clinical features at both the baseline and post-first injection time of a given patient should be obtained by AI models. The trained model will provide extendibility probability up to a certain interval, namely, overall expected treatment needs as well as optimal extension length over a definite time frame. Administered in clinical practice, these models boosted by AI will enhance the plannability of anti-VEGF treatments, such as reducing healthcare costs and managing the expectations of physicians and patients, resulting in better outcomes due to the prevention of under- or overtreatment widely.

With the advancing guidance of AI in the prognosis and therapeutics of DR, more and more stakeholders, including scientists, clinicians, regulatory agencies, and patients, consider it necessary to pave the road toward the development and implementation of an updated staging system for DR. Given the complexities of diabetic pathways and pathology, most systems may need diverse information from AI to be included in updated staging systems for DR (80). Advanced AI-based disease models will elucidate on DR pathophysiology and further explore the neglected knowledge by interpreting the microstructural features from predictive analyses.

## From diabetic retinopathy to systemic diseases

As a relatively simple, safe, validated, and acceptable routine test, retinal photography for DR screening easily accumulated to the largest medical image-level dataset. That would be an access to relevant endocrine disease or even other systemic diseases data-driven by AI technology potentially. The development of automated analysis software using AI-based deep neural learning for retinal images will allow the development of specific software to define cardiovascular risks in diabetic individuals on the basis of retinal structures as well as functional microvasculature changes (81, 82).

By ophthalmoscopy the diagnoses of various systemic diseases start to be part of eye specialists’ work, including CMV infection, hypertension, syphilis, tuberculosis, sarcoidosis, and other autoimmune diseases. For DR complication, Kang Zhang et al. (83) found that the identification of chronic kidney disease (CKD) and type 2 diabetes (T2DM) could be attained by DL models using only fundus images or multimodal information combined with clinical metadata (sex, age, height, body mass index, weight, and blood pressure) achieving the AUCs of 0.85–0.93. This study also assessed the feasibility of predicting CKD progression in a longitudinal cohort. Especially, the estimated glomerular filtration rate (eGFR), as an important biomarker to diagnose, could be accurately predicted by an AI model using their fundus images alone, expanding the scope of DR screening insightfully.

Patients with T2DM have higher than average risks for neurodegenerative disease development, especially cognitive dysfunctions, including Alzheimer’s disease (84). Since the retina is embryonically a brain-derived tissue, the eye is supposed to provide an efficient window into the brain, facilitating easy, non-invasive investigations of neurodegenerative comparisons between the brain and retina. The measurement of neuroretina or retinal fiber layer thickness by SD-OCT (85), or the evaluation of retinal sensitivity (86) as well as gaze fixation by microperimetry (87), are proven to be indicative for the identification of T2DM patients with mild cognitive impairments, which may be a prodromal sign of Alzheimer’s disease. These results elucidate on strategies for DR screening in individuals older than 60 years, as screening for retinopathy may not be limited to keeping from sight-threatening disease but may also be used to early detect the individual risk of severe cognitive decline.

It is said that eyes are the windows to the mind. Exactly, the retina is fairly special as it is the only place of the human body where vascular tissues can be rapidly and non-invasively visualized. Efforts have been aimed deeply at improving the risk prediction of cardiovascular disease (CVD), especially by integrating phenotypic features to promote and the addition of retinal imaging. CVD-associated conditions, including cholesterol emboli and hypertensive retinopathy, can often manifest in the eye. Several retinal features were used to predict cardiovascular events, such as stroke (88) or chronic kidney disease (89), previously. These specific features include vessel caliber, bifurcation, and tortuosity, which could easily be detected by advanced ML/DL. A DL model predicting cardiovascular risk factors was built by Poplin and Varadarjan et al. (90) using retinal fundus images from 48,101 patients from the UK Biobank study dominated by Caucasians without diabetes and 236,234 patients from the EyePACS population of mainly diabetic Hispanics. The validation of these models was carried out using images from 999 patients in EyePACS, 12,026 patients in UK Biobank, and an external cohort comprising Asian patients (91). The model was then established to be fairly accurate for various predictions, including age (mean absolute error ± 3.26 years), sex (AUC = 0.97), systolic blood pressure (mean absolute error within 11 mmHg), and smoking status (AUC = 0.71). In addition, a model was trained for the prediction of the onset of major adverse cardiovascular events within 5 years, achieving an AUC of 0.70 (95% CI: 0.65, 0.74) from retinal fundus images independently, which is equivalent to the AUC of 0.72 (95% CI: 0.67, 0.76) for the European SCORE risk calculator.

**Figure 4:**
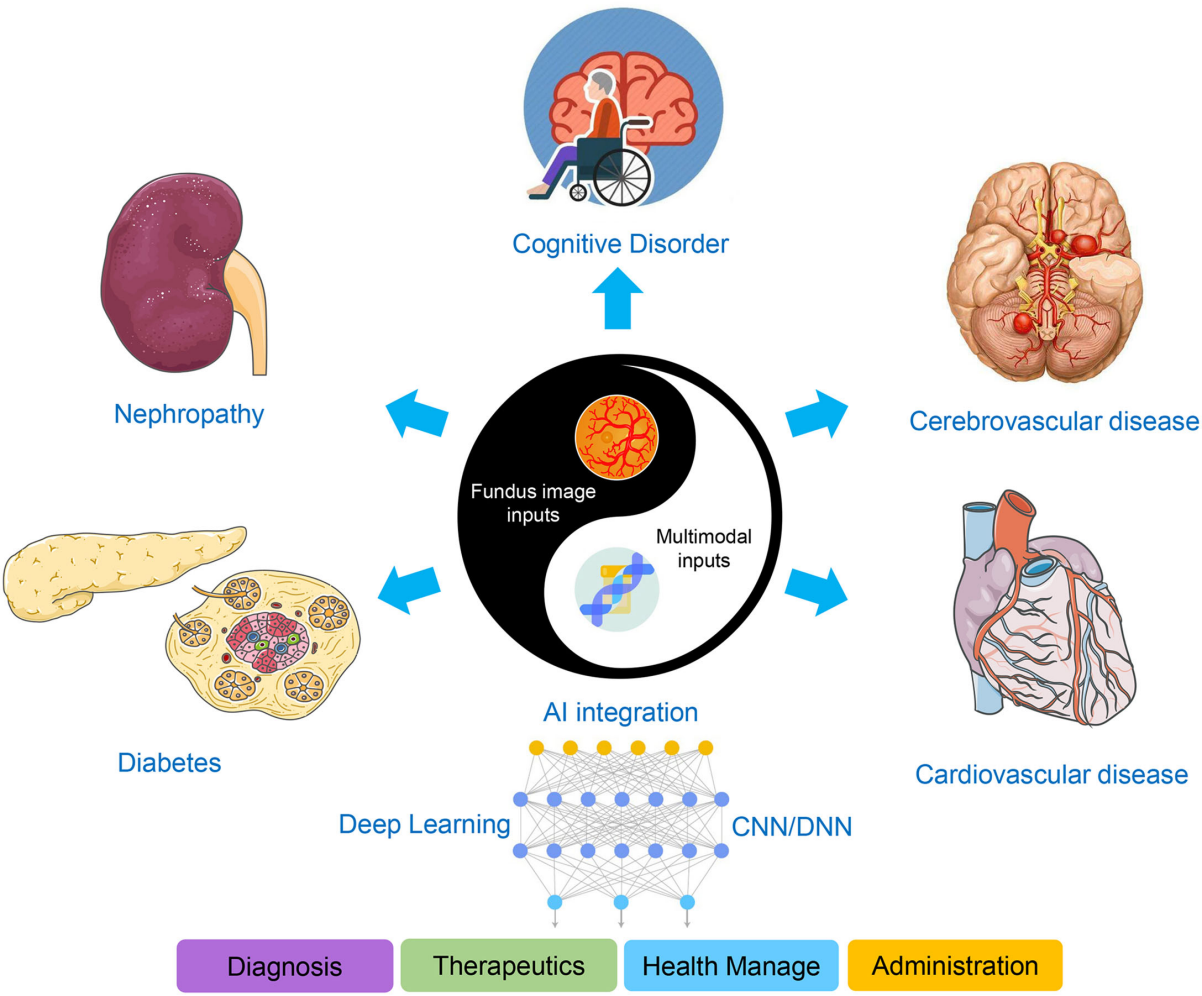
Scheme of the AI-based applications integrating DR screening with multimodal features. Fundus image inputs, including digital fundus photography and OCT, integrated with multimodal features, are indicative for diverse systemic diseases such as diabetes, nephropathy, cognitive disorder, and cerebro- or cardiovascular disease, facilitating the four perspectives of medical practice concerned.

Populations with more cardiovascular events or a markedly larger dataset will enable DL models to be more accurate, evaluated, and trained with high confidence. The use of larger datasets for training and more clinical demonstrations will inform whether retinal fundus images will augment or replace some conventional costly or invasive markers (92). DL systems have the potential of accepting multiple data types as inputs to unearth specific relevance for heterogeneous healthcare data, such as genomic profiling, time-series information, and clinical features, to yield more robust accuracy in disease identifications and predictions (**Figure 4**). As a window to health, once extensively “exploited” by AI approaches, the retina is expected to be a new focus of both non- and ophthalmological research.

## Conclusion

Translating AI to ophthalmic clinic still faces some problems in reality. Although there are many representative international datasets of DR available to build the diagnosis model, they were designed with insufficient consideration for clinical adaptability. The data quality was uneven and the labeling standards varied. Therefore, a standardized dataset with a large sample size needs to be constructed with authoritative and recognized high-quality annotations, which is prerequisite to improve the performance of AI systems finally. In addition, the regulatory mechanism and evaluation criteria have not yet formed a complete and unified system. It is also necessary to establish and improve an AI product evaluation system that conforms to medical evaluation standards before the large-scale clinical use of AI, in order to ensure its safety and effectiveness.

Since DR has seen the first FDA clearance named IDx-DR for an autonomous AI diagnostic system without an image interpretation provided by a specialist, more and more commercial products are developed and believed to come into the market in the near future. In China, training and validation data for AI algorithms are vast due to a rather centralized healthcare system and the largest population of DR (93). Hundreds of new start-up companies working on AI applications to healthcare in China have emerged to improve business, and several DR AI-based screening tools have acquired the certificate of medical device Class III approved by NMPA as pioneers, e.g., Silicon Intelligence, Airdoc, Vistel, and especially Intelligent Healthcare of Baidu that developed the first granted algorithm working robustly with various fundus camera models and achieving high accuracies for detecting multiple ophthalmic diseases (94, 95). Undoubtedly, the real-world deployment of these new systems in multiple settings will be full of challenges not only in AI diagnostic technologies but also in the marketing pattern and policy-making. The combination of telemedicine aided by 5G technology and automated retinal image analysis will enhance the convenience of DR care by providing automated real-time assessment in a more personalized way, supporting commercial interests to promote the whole industry. Additionally, multicenter, head-to-head, real-world validation, multimodality, and improved algorithm studies are imperative and encouraged to conduct, which might magnify the significance of DR screening beyond preventing sight-threatening diseases to the new strategies of systemic diagnosis in metabolism and endocrinology.

## Author contributions

XH and HW contributed to the writing of the manuscript under YT’s supervision. CS, JF, XL, XFH, and LC do data collection, reference updating and checking. All authors contributed to the article and approved the submitted version.

## Funding

National Natural Science Foundation of China (Award number(s): 32000485, 62006161, 82070948). Scientific Research Program of Beijing Municipal Commission of Education (KM202010025020) and Shunyi District “Beijing Science and technology achievements transformation coordination and service platform” construction fund (SYGX202010).

## Conflict of interest

The authors declare that the research was conducted in the absence of any commercial or financial relationships that could be construed as a potential conflict of interest.

## Publisher’s note

All claims expressed in this article are solely those of the authors and do not necessarily represent those of their affiliated organizations, or those of the publisher, the editors and the reviewers. Any product that may be evaluated in this article, or claim that may be made by its manufacturer, is not guaranteed or endorsed by the publisher.
